# The Association between Deoxyribonucleic Acid Hypermethylation in Intron VII and Human Leukocyte Antigen-C*∗*07 Expression in Patients with Endometriosis

**DOI:** 10.1155/2023/2291156

**Published:** 2023-03-18

**Authors:** Wenrong Zhao, Lei Lei, Rui Chen, Yanmin Zhang, Linlin Chang, Jingxin Cheng

**Affiliations:** Department of Obstetrics and Gynecology, East Hospital, School of Medicine, Tongji University, Shanghai 200120, China

## Abstract

**Objective:**

Endometriosis, which is a common disease affecting approximately 10% of women of reproductive age, usually causes dysmenorrhea and infertility, thus seriously harming the patients' physical and mental health. However, there is a mean delay of 6.7 years between the onset of the symptoms and the surgical diagnosis of endometriosis. There is an increasing amount of evidence that suggests that epigenetic aberrations, including deoxyribonucleic acid (DNA) methylation, play a definite role in the pathogenesis of endometriosis. This study aimed to explore the noninvasive or minimally invasive biomarkers of this disease.

**Materials and Methods:**

Six patients with surgically confirmed ovarian endometriosis and six patients who received IUD implantation for contraception without endometriosis were recruited in the East Hospital of Tongji University in 2018. The genome methylation profiling of the eutopic and ectopic endometrium of ovarian endometriosis patients and normal endometrial specimens from healthy women was determined using a methylation microarray test. The test screened methylation-differentiated 5′–C–phosphate–G–3′ (CpG) sites and then located the target genes affected by these sites following sequence alignment. Then, an additional 22 patients and 26 healthy controls were enrolled to further verify the difference in the selected genes between endometriosis patients and healthy women. The differential DNA methylation of the selected genes was validated via direct bisulfite sequencing and analysis of their messenger ribonucleic acid (mRNA) levels using quantitative reverse transcription polymerase chain reaction (qRT-PCR).

**Results:**

Fifteen differentially methylated CpG sites were found among the patients and healthy women, and five CpG sites were mapped to the introns of the human leukocyte antigen-C (HLA-C) gene; these were highly polymorphic between different HLA-C alleles and were HLA-C*∗*07 specific. The results indicated that the HLA-C*∗*07 carrier patients exhibited significantly higher DNA methylation levels at the intron VII of HLA-C compared to the HLA-C*∗*07 carrier healthy controls. High HLA-C*∗*07 mRNA levels were also observed using qRT-PCR with HLA-C*∗*07-specific primers, which indicated that the hypermethylation of CpG in intron VII might suppress a silencer that regulates HLA-C*∗*07 expressions.

**Conclusion:**

Deoxyribonucleic acid hypermethylation in the intron VII of the HLA-C*∗*07 gene appears to regulate the expression of HLA-C*∗*07. The aberrant DNA methylation in this region was positively correlated with the occurrence of endometriosis.

## 1. Introduction

Endometriosis is a chronic gynecologic disease that affects approximately 10% of women of reproductive age [1]. The disease is typically characterized by the presence and growth of endometrial epithelium and stroma outside the uterus, primarily in the pelvic peritoneum and on the ovaries [2]. Endometriosis is a benign disease, however, it is aggressive, with distant metastases and a high recurrence rate. Its high prevalence and severe complications, which include infertility and pelvic pain, make this disease a major public health concern.

The etiology and pathogenesis of endometriosis require further investigation. The existing theories on the cellular origin of endometriosis include retrograde menstruation, coelomic metaplasia, embryonic cell rest theory, and stem cell theory [3, 4]. The occurrence and severity of endometriosis are influenced by hormonal levels, genetic background, and immune response, as well as certain environmental and lifestyle factors [5]. Recent evidence suggests that various epigenetic modifications play an essential role in the etiology and pathogenesis of endometriosis [6].

Deoxyribonucleic acid (DNA) methylation is a widely understood epigenetic modification, and aberrant DNA methylation represents a potential mechanism of endometriosis [7–9]. Ectopic endometrial tissues express variable levels of DNA methyltransferase enzymes, which introduce and maintain DNA methylation on the C5 position of cytosine in 5′–C–phosphate–G–3′ (CpG) dinucleotides [10]. Abnormal DNA methylation in endometriosis then affects the expression of several genes, including homeobox A10 [11–13], estrogen receptor [14], progesterone receptor [15], steroidogenic factor 1 (SF-1) [16, 17], and aromatase [18].

It has been estimated that there is a mean delay of 6.7 years between the onset of the symptoms and the surgical diagnosis of endometriosis [19, 20]. Many studies have been conducted to identify novel diagnostic markers. Although the immunologic (IL-6 [21], TGF-*β* [22], etc.), genetic (miR-200 family [23], exosomal miR-215-5p [24], etc.), and heterogeneous (CA125 [20], leptin [25], etc.) biomarkers may be helpful in the diagnosis of endometriosis, most of them are not highly specific and can be changed in many other pathological conditions such as inflammation and malignancy. In addition, many markers are not sensitive enough in patients with early endometriosis [26]. For example, CA125 is the most valuable marker in clinical application. CA125 ≥ 30 U/mL can highly predict female patients with pain and/or infertility, but CA125 < 30 U/mL cannot exclude endometriosis [20]. Therefore, CA125 can be used as a routine test in symptomatic patients, but is not recommended as a screening marker. Thus, there is an urgent need for novel potential biomarkers. This study involves the screening of several epigenetic candidates as biomarkers for endometriosis and evaluates the effect of DNA methylation on gene expression.

## 2. Materials and Methods

### 2.1. Study Design

#### 2.1.1. Microarray Study

To screen and locate the genome methylation sites. Patients with ovarian endometriosis and healthy women were recruited in a small sample in the Shanghai East Hospital from 2018.7.1–2018.10.31. There were 21 eligible patients with ovarian endometriosis, and finally, 6 women were willing to participate while 6 of 48 eligible healthy women joined. The methylation-differentiated 5′–C–phosphate–G–3′ (CpG) sites screening of the eutopic and ectopic endometrium of ovarian endometriosis patients and normal endometrial specimens from healthy women was determined using a methylation microarray test. Then we located the target genes affected by these sites following sequence alignment.

#### 2.1.2. Validation Study

To further verify the difference of the selected genes between patients with ovarian endometriosis and healthy women.

An additional 22 patients and 26 healthy controls were enrolled in the same hospital during 2019.2.1–2020.1.31. The differential DNA methylation of the selected genes was validated via direct bisulfite sequencing and analysis of their messenger ribonucleic acid (mRNA) levels using quantitative reverse transcription polymerase chain reaction (qRT-PCR).

We nonselectively enrolled eligible patients who were willing to participate in both two cohorts.

### 2.2. Participants

Diagnostic criteria were as follows: (1) patients with ovarian endometriosis were surgically and pathologically confirmed and (2) healthy women were selected those people who were about to receive IUD implantation for contraception without dysmenorrhea, infertility, or chronic pelvic pain, while no ovarian cyst detected by gynecological ultrasound, and serum CA125 was normal.

Inclusion criteria were as follows: (1) women aged 20 to 45; (2) with regular menstrual cycle within one year; (3) good mental state; and (4) willing to participate.

Exclusion criteria were as follows: (1) took oral sex hormones within 3 months; (2) with a history of the pelvic inflammatory disease; (3) women in menstrual period or pregnant; (4) with a history of the gestational trophoblastic disease; (5) women in menstrual period or pregnant; (6) suspected or previously suffered from cancer; and (7) suspected or previously suffered from other chronic diseases, such as diabetes, hypertension, hyperlipidemia, and immunological diseases.

### 2.3. Human Tissue Collection

Eutopic endometrium from disease-free participants and patients with endometriosis was obtained at the time of laparoscopy, and ectopic endometrium from the cyst walls of ovarian endometriomas was obtained immediately after surgery. Endometriosis was confirmed for each sample via histological examination. The general characteristics of the participants are listed in Table 1.

### 2.4. Deoxyribonucleic Acid Isolation and Bisulfite Treatment

The total genomic DNA from each sample was isolated using a QIAamp Fast DNA Tissue Kit (Qiagen, Valencia, California, the United States), and the DNA quality was assessed via visualization following agarose gel electrophoresis. To selectively convert unmethylated cytosine to uracil, 1 *µ*g of the genomic DNA was subjected to bisulfite treatment using EZ DNA Methylation kits (Zymo, Orange, California, the United States).

### 2.5. Beadchip Arrays

The converted genomic DNA was hybridized to the Infinium Human Methylation 450 BeadChip assay and scanned using the Illumina iScan system, while the image data were processed using Genome Studio software. Methylated and unmethylated signals were used to compute beta (*β*) scores, which are quantitative scores of DNA methylation levels, ranging from 0 (indicating completely unmethylated) to 1 (indicating completely methylated).

### 2.6. Human Leukocyte Antigen-C*∗*07 Typing

Human leukocyte antigen (HLA)-C*∗*07 typing was performed using the polymerase chain reaction (PCR) method with HLA-C*∗*07-specific primers (forward, 5′-AGAAAGCAGAAGTCCTTCT-3′ and reverse, 5′-TACATCCGTCCTTCATTGTCA-3′). The PCR was carried out in a thermocycler at 94°C for 3 min, followed by 30 cycles of denaturation at 94°C for 30 s, annealing at 55°C for 30 s, elongation at 72°C for 1 min, and, finally, extension at 72°C for 3 min. The amplified PCR products were separated in 1% agarose gel and visualized using ethidium bromide staining.

### 2.7. Bisulfate Sequencing Polymerase Chain Reaction

The bisulfite sequencing primers (forward, 5′-TGTGGTGGGTYGTTTAGAGTGTTATT-3′ and reverse, 5′-AATAAACRATAACACTCTAAACAACC-3′) for HLA-C*∗*07 intron VII amplified a 298-base pair product flanking eight CpG dinucleotides. The PCR conditions were 94°C for 3 min, followed by 40 cycles of denaturation at 94°C for 1 min, annealing at 52°C for 2 min, elongation at 72°C for 1 min, and then extension at 72°C for 10 min. The PCR products were cloned in a pGEM-T Easy vector (Promega, Madison, Wisconsin, the United States). Following transformation, 20 individual clones were selected at random, and 10 positive clones containing the correct insert were then sequenced using an Applied Biosystems 377 instrument. The sequence data were analyzed using Molecular Evolutionary Genetics Analysis version 5 software.

### 2.8. Reverse-Transcription and Quantitative Polymerase Chain Reaction

The total ribonucleic acid (RNA) was isolated using TRIzol™ (Invitrogen, the United States), according to the manufacturer's protocol. A 1-*µ*g volume of total RNA was used to generate complementary DNA (cDNA) with the Superscript III first-strand synthesis system (Invitrogen, Carlsbad, California, the United States). Real-time quantitative PCR was performed using a SYBR green mix on an ABI 7900 detection system (Applied Biosystems, Massachusetts, the United States). The relative gene expression was assessed using glyceraldehyde-3-phosphate dehydrogenase (GAPDH) as a reference gene. The following primers were used for the HLA-C*∗*07 coding region: forward, 5′-CGGGATGGGGAGGACCAGACCC-3′ and reverse, 5′-CATAGCGGTGACCACAGCTCCA-3′, while the GAPDH primers were forward, 5′-GGAGCGAGATCCCTCCAAAAT-3′ and reverse, 5′-GGCTGTTGTCATACTTCTCATGG-3′.

### 2.9. Data Analysis

For the microarray study, the differences in CpG methylation sites were determined with a false discovery rate of <0.05, *P*  <  0.05, and Δ*β* > 0.04, while for the validation study, the percentage methylation of each clone obtained from every individual was treated as a single value for the statistical analysis of the bisulfite sequencing. The data were analyzed using a Student's *t*-test to compare the percentage methylation of the two groups, with a statistical significance set at the level of *P*  <  0.05

## 3. Results

### 3.1. Differential Screening of the 5′–C–Phosphate–G–3′ Methylation Sites in the Endometrial Tissues of Patients and Healthy Women

**Figure 1:**
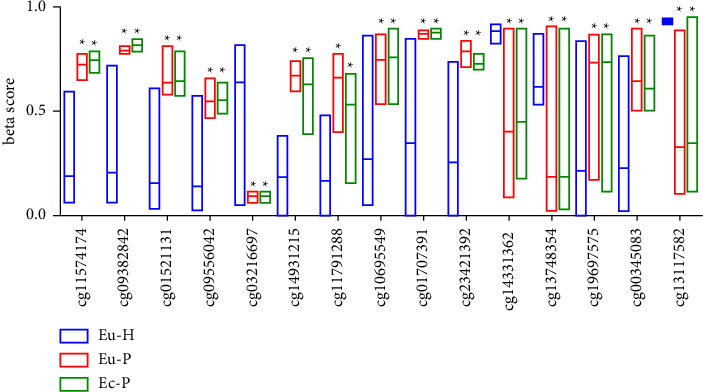
Differentially methylated 5′–C–phosphate–G–3′ (using 450K arrays) of the eutopic endometrium of healthy women and the eutopic and ectopic endometrium of patients with endometriosis; Eu-H is the eutopic endometrium of healthy women; Eu-P is the eutopic endometrium of patients with endometriosis; and Ec-P is the ectopic endometrium of patients with endometriosis (^*∗*^*P* <  0.05).

The methylation profiles of ectopic and eutopic endometrium from the endometriosis patients were compared with the eutopic endometrium of healthy women. The 15 common sites of comparison are shown in Figure 1. No significant differences were observed in the methylation profiles of the 15 sites with respect to the ectopic tissues, suggesting that these sites may be potential diagnostic markers of endometriosis; there were only significant differences in the methylation profiles of the patients and the healthy controls.

Of the 15 differentially methylated CpG sites, 11 were hypermethylated in the patients, while the other four sites were hypomethylated. All the sites were then mapped to the genome sequence (Table 2). Ten sites were located in different genes or loci, but the other five CpG sites (cg03216697, cg01521131, cg09382842, cg11574174, and cg09556042) were mapped to the same gene, namely, HLA-C. The HLA-Cgene-specific CpG sites were hypermethylated in the endometriosis patients, except for cg03216697.

### 3.2. Human Leukocyte Antigen-C*∗*07 Typing

**Figure 2:**
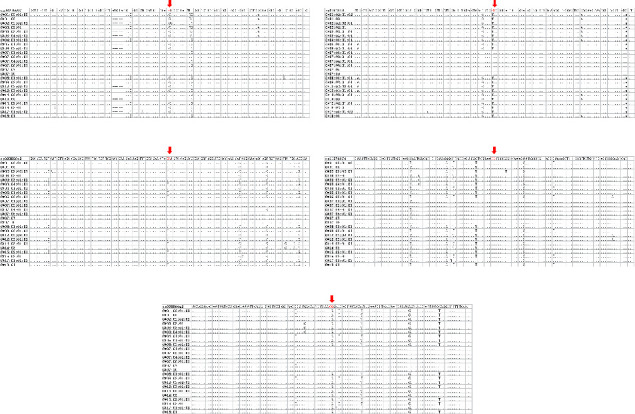
The deoxyribonucleic acid (DNA) alignment of five aberrantly methylated 5′–C–phosphate–G–3′ (CpG) sites to different human leukocyte antigen-C (HLA-C) alleles. Twenty-six common HLA-C alleles were selected for alignment. The dots indicate the same DNA sequence as that present in the first line. The target CpGs are highlighted with red arrows.

A potential bias in estimating DNA methylation with 450K arrays can arise from the impact of single nucleotide polymorphisms. This is of particular importance in the HLA region due to the high density of polymorphic sites found there and the sequence similarity among proximal HLA genes. Following sequence alignment (Figure 2), the CpG sites of cg03216697, cg01521131, cg09382842, and cg09556042 were found in the HLA-C*∗*07 allele cluster, but they were abolished in the other HLA-C alleles although the CpG site of cg11574174 was conserved in all the HLA-C alleles.

**Figure 3:**
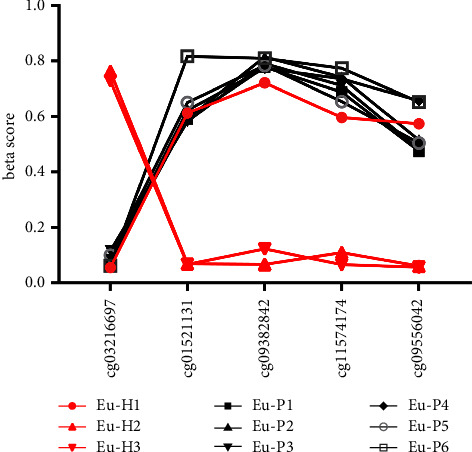
The beta scores of five aberrantly methylated 5′–C–phosphate–G–3′ sites mapped to the human leukocyte antigen-C (HLA-C) gene in HLA-C*∗*07 carriers. Following HLA-C genotyping, nine HLA-C*∗*07 carriers were selected from the 12 participants in the microarray study. The deoxyribonucleic acid methylation status of the healthy woman (Eu-H1) was identical to that of the patients (Eu-P1–Eu-P6), while the other two (Eu-H2 and Eu-H3) were different.

To rule out the effects of HLA polymorphisms on the methylation microarray test, the HLA genotypes of the 12 participants evaluated in the microarray were analyzed. Six patients were found to be HLA-C*∗*07 carriers, while there were only three such carriers in the healthy control group. This result indicates that the differential methylation level of HLA-C may be due to genetic polymorphism. Nevertheless, it was observed that of the three healthy HLA-C*∗*07 carriers, one patient had a methylation profile similar to that of the endometriosis patients, but the other two had markedly different profiles (Figure 3). It was therefore assumed that the methylation profile of HLA-C*∗*07 in endometriosis patients and healthy women is different.

### 3.3. Validation of the Differential Deoxyribonucleic Acid Methylation of Human Leukocyte Antigen-C*∗*07 by Direct Bisulfite Sequencing

**Figure 4:**
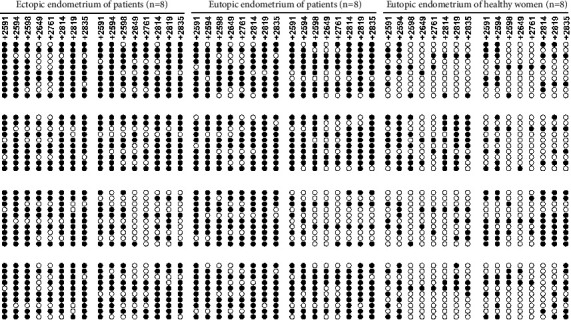
The deoxyribonucleic acid methylation status of human leukocyte antigen-C (HLA-C)*∗*07 introns VII. The open and filled circles represent unmethylated and methylated cytosines, respectively. The numbers on top indicate the positions of cytosine residues of 5′–C–phosphate–G–3′ relative to the transcriptional start site (+1). A total of 16 HLA-C*∗*07-carrier individuals were enrolled: eight patients with endometriosis and eight healthy women. Both the eutopic and ectopic endometria of the patients were analyzed.

To further verify the DNA methylation difference of HLA-C*∗*07 between endometriosis patients and healthy women, an additional 22 patients and 26 healthy controls were enrolled in the study, and the HLA genotypes of all 48 were typed. Eight HLA-C*∗*07 carriers in the patient group and eight in the healthy control group were selected for validation analysis, which focused on the intron VII of HLA-C*∗*07 since two adjacent differentially hypermethylated CpG sites (cg09382842 and cg11574174) were mapped to this region. The intron VII of HLA-C*∗*07 was then amplified using an HLA-C*∗*07-specific bisulfate sequencing PCR following bisulfate modification. The methylation profiles of the ectopic and eutopic endometrium of the patients were similar, but both were significantly different from the eutopic endometrium profiles of the healthy women (Figure 4). The ectopic and eutopic endometrium of the patients presented a dense methylation pattern in the intron region, while most of the CpG sites were not methylated in the eutopic endometrium of the healthy women. Of the 8 HLA-C*∗*07 endometriosis patients, 87.5% (7/8) of patients exhibited significantly higher DNA methylation levels in the intron VII of the HLA-C gene, compared to 25.0% (2/8) in healthy women.

### 3.4. High Expression of Human Leukocyte Antigen-C*∗*07 in Endometriosis Patients

**Figure 5:**
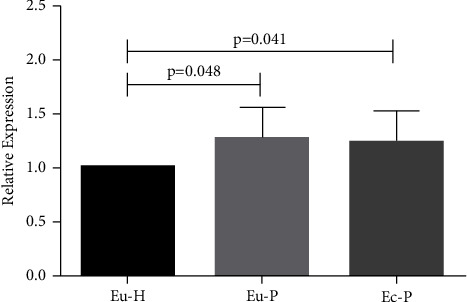
The relative messenger ribonucleic acid (mRNA) expression of human leukocyte antigen-C (HLA-C)*∗*07 in patients and healthy women. The relative mRNA expression of HLA-C*∗*07 was detected using a quantitative reverse transcription polymerase chain reaction with HLA-C*∗*07-specific primers. Eu-H is the eutopic endometrium of healthy women; Eu-P is the eutopic endometrium of patients with endometriosis; and Ec-P is the ectopic endometrium of patients with endometriosis.

To characterize the effects of DNA methylation at the intron VII region on HLA-C*∗*07 gene expression, the messenger ribonucleic acid (mRNA) levels of the HLA-C*∗*07 carriers were analyzed using reverse transcription qPCR. As shown in Figure 5, the expression levels of HLA-C*∗*07 in the patients were found to be slightly, but significantly, higher than the levels in the healthy carriers.

## 4. Discussion

While the pathogenesis of endometriosis is not clear, an increasing number of studies have confirmed that it may be an epigenetic disease. Deoxyribonucleic acid methylation, which affects the occurrence and development of diseases by regulating gene transcription, is the most common modification mode in epigenetics The acquisition of abnormal methylation patterns in patients with endometriosis through noninvasive methods may help in exploring the pathogenesis of the disease and searching for new diagnostic markers and new treatments.

Using a methylation microarray, this study found 15 CpG sites that were differentially methylated in patients with endometriosis and healthy women. Five CpG sites mapped to HLA-C, an HLA class I gene, were confirmed as HLA-C*∗*07 specific. The frequency of intron VII methylation for HLA-C*∗*07 in the endometrial tissues of the endometriosis patients was significantly higher than that of the healthy controls, as detected via bisulfite sequencing. There was no significant difference in HLA-C*∗*07 methylation between the eutopic and ectopic endometrium of the patients. This finding suggests that HLA-C*∗*07 intron VII hypermethylation may be an epigenetic marker for endometriosis. This special methylation pattern is likely to be involved in the onset of endometriosis, but this requires further investigation.

Genetic variation in HLA genes is of important clinical significance since it is associated with susceptibility to autoimmune and infectious diseases and plays a major role in both transplantation and cancer. The expression of HLA class I has been found to be higher in ectopic endometrial tissues when detected via immunohistochemistry [27], and HLA class I expression appears to be induced by interferon and tumor necrosis factor in normal endometrium [28, 29]. However, the levels of these cytokines in the endometrium are similar in women with or without endometriosis [30]. In recent studies, the methylation status of HLA promoters was investigated in terms of esophageal squamous cell carcinomas [31], gastric cancer [32], and psoriasis [33], and the HLA promoter was observed to be hypomethylated or nonmethylated in the healthy controls. While the hypermethylation of promoters leads to HLA class I antigen downregulation in cancer, the aberrant promoter methylation cannot explain the high expression of HLA in endometriosis and HLA-C in psoriasis [33]. The current study found that the hypermethylation of intron VII led to the high expression of HLA-C*∗*07 in women with endometriosis.

Deoxyribonucleic acid methylation at the promoter region is a widely accepted epigenetic mechanism affecting gene expression. However, the binding sites for transcription factors might be a great distance from the transcriptional start sites [34], and those located in introns or those that are distal to genes might interact with promoters via looping chromatin [35, 36]. Xue et al. reported that the hypermethylation of CpG islands spanning exon II to intron III activated SF-1 gene mRNA expression in endometriosis [17]. The present study observed a similar mechanism with HLA-C*∗*07, and it was hypothesized that intron VII of HLA-C*∗*07 might enclose a silencer that is suppressed when hypermethylated, leading to increased HLA-C*∗*07 expression.

Depending on the amino acid found at position 80, HLA-C alleles can be divided into two groups, namely, C1^Asn80^ and C2^Lys80^ [37], and HLA-C*∗*07 is grouped in C1, which is the ligand of the killer cell immunoglobulin-like receptor (KIR) of natural killer (NK) cells. The interaction of C1 and KIR2DL2 or KIR2DL2 then inhibits NK cell activity [38, 39].

Sampson's hypothesis is the most widely accepted theory for explaining the origins of endometriosis [4]. According to this hypothesis, impaired NK activity in women with endometriosis is thought to promote the implantation and progression of endometrial tissue. The higher HLA-C*∗*07 mRNA expression observed in the current study partially explains the resistance of endometrium to NK cells. We hypothesized that the intron VII of HLA-C*∗*07 might contain a silencer that downregulates the expression of HLA-C*∗*07 (a ligand for the inhibitory KIR signal on the surface of NK cells). Due to the low expression of HLA-C*∗*07 in the healthy group, NK cells can normally clear the endometrial tissue that is countercurrent to the pelvic cavity with menstrual blood, reducing the risk of endometriosis. However, the hypermethylation in an intron of HLA-C*∗*07 could relieve the inhibition of HLA-C*∗*07 expressions, and then by acting on KIR, inhibit the activity of NK cells and promote the occurrence of endometriosis. Of course, this hypothesis needs further study in the future.

Due to the lack of sensitive and specific biomarkers in clinical practice, many patients are already at the advanced stage of the disease when they are diagnosed with endometriosis. This research indicated that multiple abnormal DNA methylation sites in patients with endometriosis were HLA-C*∗*07 specific, while the HLA-C*∗*07 carrier patients exhibited significantly higher DNA methylation levels at the intron VII of HLA-C compared with the HLA-C*∗*07 carrier healthy controls. This may present a new method for the early and noninvasive clinical diagnosis of endometriosis.

The hypermethylation of CpG in the intron VII of HLA-C*∗*07 was associated with high HLA-C*∗*07 mRNA levels, which could be attributed to the suppression of a silencer that regulates HLA-C*∗*07 expressions. This finding contributes to the current understanding of the pathogenesis of endometriosis.

It is worth mentioning that in the microarray study, we nonselectively enrolled eligible patients who were willing to participate. To our surprise, all 6 patients in the microarray study were coincidentally HLA-C*∗*07 carriers, not due to any subjective selection behavior by the investigators. Then it seems that the allele frequency of HLA-C*∗*07 in endometriosis patients (6/6, 100%) was higher than in healthy controls (3/6, 50%). However, the allele frequencies of HLA-C*∗*07 in endometriosis patients (36.4%) and healthy controls (30.1%) were similar in the validation study.

This study involved a number of limitations. First, the number of HLA-C*∗*07 carriers evaluated was relatively small and additional studies are needed to support the conclusions. Second, since HLA-C genes are highly variable, the methylation statuses of other alleles also need to be investigated. Finally, the test requires the acquisition of organization and loses the advantage of noninvasiveness. The finding is still a long way from being a diagnostic marker for endometriosis.

## 5. Conclusions

In conclusion, DNA hypermethylation in the intron VII of the HLA-C*∗*07 gene appears to regulate the expression of HLA-C*∗*07. The aberrant DNA methylation in this region was positively correlated with the occurrence of endometriosis. This finding may be of more significance to advance the research on the immunopathological mechanism of endometriosis.

## Figures and Tables

**Table 1:** General characteristics and HLA-C typing of the study participants.

Microarray study	Patients with ovarian endometriosis (*n* = 6)	Healthy controls (*n* = 6)
Age (years, mean ± SD)	33.4 ± 4.1	32.5 ± 3.6
BMI (kg/m^2^, mean ± SD)	24.2 ± 3.2	25.1 ± 2.9
Stage I-II (*n*)	4	NA
Stage III-IV (*n*)	2	NA
*HLA-C typing*		
HLA-C*∗*07 carrier (*n*)	6	3
Non-HLA-C*∗*07 carrier (*n*)	0	3

Validation study	Patients with ovarian endometriosis (*n* = 22)	Healthy controls (*n* = 26)
Age (years, mean ± SD)	32.3 ± 3.8	32.9 ± 4.3
BMI (kg/m^2^, mean ± SD)	22.2 ± 3.9	24.3 ± 3.4
Stage I-II (*n*)	16	NA
Stage III-IV (*n*)	6	NA
*HLA-C typing*		
HLA-C*∗*07 carrier (*n*)	8	8
Non-HLA-C*∗*07 carrier (*n*)	14	18

NA, not applicable.

**Table 2:** Fifteen aberrantly methylated CpG sites and the mapped gene.

No	Gene
cg11574174	HLA-C
cg09382842	HLA-C
cg01521131	HLA-C
cg09556042	HLA-C
cg03216697	HLA-C
cg14931215	KDM5D
cg11791288	LOC284551
cg10695549	PSD3
cg01707391	—
cg23421392	PDIA6
cg14331362	—
cg13748354	NUDT1
cg19697575	—
cg00345083	AJAP1
cg13117582	—

## Data Availability

The data that support the findings of this study are available from the corresponding author upon reasonable request.
